# A Physician-Driven Patient Safety Paradigm: The “Pitfall Bank” as a Translational Mechanism for Medical Error Prevention

**DOI:** 10.3390/healthcare13172248

**Published:** 2025-09-08

**Authors:** Gerd Herold, Viktoras Justickis, Vytė Maneikienė, Kazimieras Maneikis, Paulius Trinkauskas, Karina Palkova

**Affiliations:** 1 Independent Researcher, Bernhard Falk Strasse 27, D50737 Cologne, Germany; gerdharaldherold@aol.com; 2 Law School, Mykolo Romeris University, 20 Ateities, LT-08303 Vilnius, Lithuania; 3 Clinic of Cardiac and Vascular Diseases, Institute of Clinical Medicine, Faculty of Medicine, Vilnius University, LT-01513 Vilnius, Lithuania; 4 Clinic of Hematology and Oncology, Institute of Clinical Medicine, Faculty of Medicine, Vilnius University, LT-01513 Vilnius, Lithuania; kazimieras.maneikis@gmail.com; 5 Faculty of Social sciences, Rīga Stradiņš University, 16 Dzirciema Street, LV-1007 Rīga, Latvia; karina.palkova@inbox.lv

**Keywords:** medical errors, patient safety, translational medicine, clinical pitfalls, Safety-I, Safety-II

## Abstract

**Background:** Despite more than 25 years of intensive effort following the landmark “To Err Is Human” report, conventional top-down medical error prevention strategies, grounded in the Safety-I paradigm, have largely failed to reduce patient harm. This persistent shortcoming underscores the need for a new prevention model. The medical literature contains an extensive yet systematically underutilized body of physician-generated experiential knowledge on “clinical pitfalls”—specific high-risk scenarios in which errors are likely to occur. This resource presents an opportunity for a novel, physician-driven approach to medical error prevention. The present paper proposes and evaluates such a model, grounded in the principles of Safety-II and translational medicine. **Methods:** The methodology involved a three-part conceptual analysis: (1) a critical review of the literature assessing the effectiveness of established error prevention strategies, (2) a quantitative bibliometric analysis of the PubMed database to determine the volume and temporal trends of publications on “clinical pitfalls”, and (3) a conceptual synthesis to design a novel physician-driven error prevention model. Each method is described in detail at the beginning of its respective section. **Results:** The literature review confirms the limited effectiveness of existing top-down safety initiatives, particularly in complex domains such as diagnosis and treatment. The bibliometric analysis identified more than 43,000 publications containing the keyword “pitfall,” with a sustained and significant upward trend in annual publications over the past three decades. The conceptual synthesis demonstrates that a physician-driven system—centered on a “Pitfall Bank”—addresses core weaknesses of current strategies, including unreliable data, heterogeneous knowledge, and cognitive biases. Structured as a circular translational mechanism, the proposed system facilitates a continuous cycle of practice-based problem identification and science-informed solution implementation. **Conclusions:** A physician-driven prevention system, architected as a translational engine, offers a promising and sustainable strategy to overcome the current impasse in medical error reduction and create a more resilient and adaptive healthcare system.

## 1. Introduction: The Persistent Challenge of Medical Errors

The landmark 2000 Institute of Medicine report To Err Is Human: Building a Safer Health System, marked a watershed moment in global healthcare, reframing medical errors from instances of individual negligence to complex, systemic phenomena with multifactorial causes [1]. In the quarter-century since its publication, healthcare systems worldwide have invested vast resources into a wide array of patient safety initiatives [2,3]. Yet, despite these extensive and costly efforts, the ambitious goal of substantially reducing medical errors remains largely unmet; indeed, some evidence suggests that error rates may even be increasing. A comprehensive “review of reviews” published in 2022, summarizing 76 evaluations of prevention strategies, concluded that patient safety has not measurably improved over the past 15 years, and that global initiatives have failed to produce substantial change [4]. This was confirmed by the following (2024) review, in which the insufficient effectiveness of preventive measures was established based on an analysis of publications up to 2024 [3]. This persistent, decades-long challenge underscores the urgent need to re-examine the foundational approach to patient safety.

The dominant strategy to date has been grounded in what safety scientists describe as the Safety-I paradigm—a reactive, top-down model that seeks to identify and eliminate failures after they occur [5]. This approach has led to the widespread implementation of standardized tools such as surgical checklists, computerized physician order entry (CPOE) systems, and rigid administrative protocols [6]. While these interventions have achieved isolated successes in preventing errors within simple, highly structured tasks, they have proven markedly less effective in the complex, dynamic, and cognitively demanding domains of diagnosis and treatment [7,8]. Their limitations have fueled the search for alternative frameworks.

One such alternative is the Safety-II paradigm, which shifts the focus from analyzing what goes wrong to understanding why things almost always go right [9]. This perspective emphasizes that resilience in complex systems such as healthcare arises not from rigid adherence to protocol but from empowering frontline clinicians who continually adapt to unpredictable and variable conditions. This reframing creates an opportunity to harness a vast yet systematically underutilized resource: the collective experiential knowledge of physicians, often documented in the form of “clinical pitfalls”.

This paper addresses a critical gap in both the literature and prevailing patient safety strategies by proposing and analyzing a novel, physician-driven medical error prevention model grounded in the principles of Safety-II and translational medicine. Central to this approach is the concept of a centralized, online Pitfall Bank—a dynamic, curated repository of physician-reported pitfalls and peer-reviewed prevention strategies. The contribution of this work lies in conceptualizing the Pitfall Bank not as a static archive, but as a circular translational mechanism that actively bridges the persistent gap between frontline clinical practice and formal scientific research. The analysis presented here demonstrates how this alternative model directly addresses the principal shortcomings of current top-down approaches and offers a necessary, sustainable path toward overcoming the long-standing stagnation in medical error reduction.

## 2. The Prevailing Paradigm: A Critical Analysis of Safety-I

This section will be based on a critical review of the literature on Safety-I.

A critical review of the literature is a methodical and evaluative examination of existing scholarly work on a specific topic. It goes beyond simply summarizing sources; it involves a deep analysis, synthesis, and critique of the literature to identify its strengths, weaknesses, and gaps in knowledge. This process helps to situate new research within the existing body of work and provides a foundation for advancing understanding [10].

For the past two decades, the dominant approach to patient safety—known as Safety-I—has defined safety as the absence of adverse events and sought to achieve it by eliminating errors and malfunctions. Its core logic is reactive: when an accident occurs, an investigation identifies the cause—often a human error or technical failure—and then implements a barrier or constraint to prevent recurrence. This has led to an emphasis on standardization, checklists, and rigid protocols aimed at reducing performance variability. While well-intentioned, this paradigm is built on assumptions that are increasingly ill-suited to the complexity of contemporary healthcare.

Failure 1: Unreliable and Incomplete Data

Safety-I relies heavily on institutional error-reporting systems to identify problems and inform interventions. However, substantial research shows that these systems are plagued by chronic underreporting, largely due to a pervasive “culture of silence” and clinicians’ legitimate concerns about blame, reputational harm, and litigation [11]. Even when errors are reported, documentation is frequently biased or incomplete, as physicians may “chart defensively” to minimize personal or institutional liability. Consequently, the resulting data provide a distorted and partial view of error causation, limiting the effectiveness of targeted prevention strategies. Moreover, such data are often lagging indicators of catastrophic failures rather than leading indicators of emerging risks.

Failure 2: Inability to Manage Complexity

A second major limitation of Safety-I lies in its implicit assumption that healthcare systems are decomposable and predictable—such that preventing failures of individual components will ensure overall system safety [12]. This linear, cause-and-effect logic is effective for simple, technical tasks. For instance, a surgical checklist can reliably prevent wrong-site surgery because the procedure is highly structured and involves limited variables [12].

However, this model fails in the face of clinical complexity. Diagnosis and treatment are not linear processes; they are complex, adaptive, and emergent, shaped by numerous interacting variables, incomplete information, and significant uncertainty [8]. In such contexts, adverse events often result not from a single failure but from the “functional resonance” of normal performance variability throughout the system—a non-linear phenomenon that Safety-I tools such as root cause analysis are poorly equipped to address [13].

Failure 3: Neglect of Human Adaptation and Resilience

Safety-I tends to view human performance variability as a liability to be constrained. Its reliance on protocols and standardization reflects a desire to ensure clinicians behave according to predefined “work-as-imagined” models [14]. This perspective overlooks a fundamental reality of complex work: outcomes go right not because individuals rigidly follow procedures but because they continually adapt to the unpredictable conditions of real-world practice [15]. Far from being a source of risk, frontline clinicians are a source of resilience, and their adaptive capacities are essential to maintaining safety. By focusing exclusively on preventing failure, Safety-I neglects to study, understand, and enhance the very capabilities that enable the overwhelming majority of successful outcomes in healthcare.

## 3. Alternative Framework: Safety-II and Resilience in Healthcare

In response to the shortcomings of the traditional paradigm, a complementary framework—Safety-II—has emerged from resilience engineering. Safety-II redefines safety from the mere absence of adverse events to the presence of positive capacities, namely a system’s ability to succeed under both expected and unexpected conditions. Its central premise is that the same factors that lead to failures also underlie successes; both are manifestations of performance variability [9].

Safety-II is underpinned by four core principles particularly well suited to modern healthcare:

- Healthcare as a Complex Adaptive System: Safety-II recognizes that healthcare is not a simple, linear machine but a complex adaptive system characterized by interconnected components, feedback loops, and emergent behaviors. In such systems, safety cannot be secured by controlling parts in isolation; it must be managed by understanding and influencing interactions across the whole system.
- Humans as a Resource for Resilience: While Safety-I treats human variability as a threat, Safety-II treats it as an indispensable resource. No set of rules can anticipate the full range of scenarios clinicians face. The capacity of frontline professionals to adapt, make trade-offs, and improvise in real time enables the system to function effectively despite uncertainty and variability.
- Understanding Work-as-Done: Safety-II distinguishes between “work-as-imagined” (idealized procedures in manuals and protocols) and “work-as-done” (the realities of practice). Safety improvement depends not on stricter enforcement of the imagined model but on a deep understanding of how successful adaptations occur and how they can be supported [14].
- Proactive Capacity Building: Safety-II shifts the goal of safety management from reactive error prevention to proactive capacity enhancement. Rather than focusing solely on rare failures, it seeks to strengthen a system’s ability to anticipate, monitor, respond, and learn, thereby improving overall resilience [16,17].

By reframing safety in terms of enabling success rather than merely preventing failure, Safety-II offers a robust theoretical foundation for developing safety initiatives that leverage—and strengthen—the expertise and adaptive skills of frontline clinicians. The key distinctions between Safety-I and Safety-II are summarized in Table 1.

## 4. An Untapped Resource: The Prevalence and Distribution of “Pitfalls” in Medical Literature: A PubMed-Based Bibliometric Analysis from 1990 to 2024

### 4.1. Introduction

In the complex and high-stakes environment of modern medicine, practitioners are guided by an ever-expanding framework of evidence-based guidelines, clinical protocols, and standardized procedures. This formal structure of knowledge is indispensable for ensuring a high baseline of care quality and safety. However, it cannot, by its very nature, encompass the full spectrum of clinical reality. The day-to-day practice of medicine is characterized by nuance, uncertainty, anatomical and physiological variability, and atypical disease presentations that defy simple categorization. It is within this gap between codified knowledge and practical application that the concept of the “pitfall” emerges as a critical element of professional discourse.

A medical pitfall is distinct from a simple error or mistake. It represents a potential source of misjudgment, a diagnostic trap, a procedural complication, or a therapeutic dead-end that a reasonably competent and careful practitioner might encounter. The term is inherently forward-looking and preventative; it describes a latent risk within a system or process rather than a realized adverse event. The literature dedicated to discussing these pitfalls serves a vital function, filling a critical void that formal guidelines cannot address. This body of work represents a vast repository of experiential, practice-based evidence, shared by clinicians to alert their peers to the subtle challenges and potential for error inherent in their shared work.

This study proposes that the extensive collection of articles discussing “pitfalls” constitutes a massive, self-organizing, and informal knowledge-sharing system. This system operates in parallel to formal continuing medical education (CME), driven by a powerful professional ethos to warn colleagues, enhance collective wisdom, and ultimately improve patient safety. The very choice of the term “pitfall” is significant. Unlike words such as “error” or “mistake,” which carry strong connotations of individual blame, incompetence, and failure, “pitfall” frames the issue as an external challenge or a tricky situation that any diligent professional could fall into. This linguistic choice depersonalizes the potential for misstep, thereby lowering the psychological and reputational barriers to open reporting. It shifts the focus from individual culpability to systemic risk awareness and shared learning, making the “pitfall” a powerful and socially acceptable vehicle for knowledge transfer in a profession where expertise and reputation are paramount. The existence of such a vast literature suggests a fundamental recognition within the medical community of the limitations of a purely top-down model of knowledge dissemination. It represents a bottom-up, peer-to-peer corrective and supplement to the formal canon, reflecting the profession’s mature capacity for self-correction and the generation of practical wisdom.

Given the apparent importance of this phenomenon, a systematic characterization of this literature is overdue. This study, therefore, aims to quantify and analyze the body of medical literature that explicitly discusses “pitfalls”. The primary objectives are (1) to determine the total volume of publications referencing the term “pitfall” in the modern medical era, (2) to analyze the prominence of the term within these articles (i.e., in the title or abstract) as a proxy for the topic’s centrality, and (3) to map the distribution of this literature across major medical specialties, identifying the clinical domains where this form of knowledge sharing is most prevalent. Through this analysis, we seek to provide the first empirical evidence of the scale and scope of this de facto knowledge system and to lay the groundwork for understanding its role in professional development and patient safety.

### 4.2. Data Source Selection

PubMed was chosen as the sole data source for this analysis due to a combination of factors that make it uniquely suited for this research question. First, PubMed is a specialized database containing primarily medical and biomedical publications. This focus ensures a high signal-to-noise ratio for a medically oriented search term, minimizing the retrieval of irrelevant articles where “pitfall” might be used in a non-clinical context (e.g., in ecology or engineering). Second, the journals indexed in PubMed, and by extension, its core component MEDLINE, undergo a stringent selection and review process by the National Library of Medicine. This curation provides an inherent baseline of scientific and ethical quality for the included literature, acting as a valuable pre-filter for the study’s corpus. Third, the comprehensive scope of PubMed, which at the time of the search contained over 38 million citations from MEDLINE and other life science journals, establishes it as the default gateway to the world’s published medical literature. Its extensive coverage ensures that the search captures a globally representative sample of relevant publications, making the findings more generalizable.

### 4.3. Search Strategy and Execution

A systematic literature search was performed in March 2024 to identify all relevant publications. The search strategy was designed to be both sensitive and specific, capturing all instances of the term while remaining focused on the intended clinical context.

The search period was defined as 1 January 1990 to March 2024. The year 1990 was chosen as a conventional starting point for several critical reasons. The late 1980s and early 1990s represent a watershed moment in medicine, marked by the consolidation of several transformative shifts. This period saw the rise and formalization of evidence-based medicine, the widespread clinical adoption of advanced cross-sectional imaging modalities like magnetic resonance imaging (MRI) and computed tomography (CT), the advent of molecular diagnostics and the polymerase chain reaction (PCR), and the rapid expansion of minimally invasive surgical techniques. Pitfalls described prior to this era would likely pertain to diagnostic tests, therapeutic strategies, and surgical procedures that are now largely obsolete. By setting the start date at 1990, the analysis deliberately focuses on the challenges, risks, and complexities that are directly relevant to contemporary medical practice, technology, and knowledge, thereby maximizing the utility of the findings for the modern physician.

The search itself was executed using the PubMed Advanced Search Builder. To establish the total number of publications mentioning the term, a broad and comprehensive query was constructed. This query was designed to identify the keyword in any searchable field, with additional clauses to specifically capture its appearance in high-impact locations like the title and abstract. The precise search logic for the specialty-specific searches involved combining the core term query with a query for the specialty name. For example, to identify publications in laboratory medicine, the search combined the “pitfall” query with a search for “Laboratory medicine” in all fields. This systematic approach was applied consistently across all targeted specialties. The detailed construction of the search queries is outlined in Table 2.

### 4.4. Search Validation and Relevance Confirmation

A critical step in any bibliometric study is to validate the precision of the search query. Keywords can have multiple meanings, and automated searches can retrieve irrelevant results, a phenomenon known as semantic noise. To address this, manual validation was performed. A key feature of PubMed is its provision of text excerpts (snippets) that show the keyword in its original context within the article’s abstract or text. This feature allows for a rapid qualitative check of relevance.

From the total set of retrieved results, a random sample of 1500 publications was generated. The text snippets for each of these 1500 results were manually reviewed to determine if the use of the word “pitfall” was directly relevant to a clinical, diagnostic, procedural, or medical management context. The review confirmed that over 99% of the sampled publications used the term in a manner directly relevant to medical practice. This exceptionally high relevance rate of over 99% is a significant finding in itself. It demonstrates that within the specialized domain of medical literature, the term “pitfall” is used with a remarkably specific, shared, and unambiguous meaning. This validates not only the precision of the search strategy but also the fundamental premise of the study: that “pitfall” is a stable and meaningful concept across the discipline, making it a robust and high-fidelity subject for large-scale quantitative analysis.

### 4.5. Results

The systematic search of the PubMed database yielded a substantial volume of literature, providing a quantitative measure of the discourse surrounding pitfalls in modern medicine. The analysis proceeded from an overall assessment of publication volume to a more granular examination of its distribution across clinical specialties.

### 4.6. Overall Publication Volume and Prominence

The analysis revealed a total of 37,295 unique publications containing the keyword “pitfall” or “pitfalls” within the defined search period of 1 January 1990 to March 2024. This massive volume of literature underscores the extent to which the identification and documentation of potential sources of error is an integral part of medical scientific communication.

To gauge the importance of this topic within the retrieved articles, a prominence analysis was conducted by examining the location of the keyword. Notably, in nearly 3000 of these publications (approximately 8.0%), the term “pitfall” appears directly in the article’s title. The title is arguably the most critical component of a scientific paper, designed to convey its core subject matter concisely. Its use in this location indicates that the discussion of pitfalls is not an incidental point but the primary focus and central thesis of the work.

Furthermore, in over 6000 publications (approximately 16.1%), the term is mentioned in the abstract or in the title. The abstract serves as a condensed summary of the article’s most important background, methods, results, and conclusions. The inclusion of “pitfall” in the abstract signifies its importance as a key discussion point, a major finding, or a critical takeaway for the reader. These data show that in 16.1% of this vast body of literature, the concept of a pitfall is considered a primary or major contribution of the paper. This finding refutes any potential characterization of these discussions as mere anecdotal asides, instead positioning them as a mainstream and high-priority activity in medical scholarship.

### 4.7. Distribution Across Key Medical Specialties

While the aggregate volume of “pitfall” literature is impressive, its distribution across different fields of medicine provides deeper insights into the specific challenges that define various clinical domains. The analysis confirmed that the phenomenon of documenting pitfalls spans the entire breadth of medicine. However, the frequency of these publications is not uniform, with certain specialties demonstrating a particularly high volume of such literature. As detailed in Table 3, surgery, histopathology, and pediatrics emerged as fields with a pronounced focus on identifying and communicating pitfalls.

This distribution is not random; it appears to reflect the unique nature of the risks and challenges inherent to each field. The high concentration of “pitfall” literature in surgery, histopathology, and pediatrics, for example, points toward distinct archetypes of medical risk. Surgery is a domain fundamentally defined by procedural and anatomical risk. The practice involves direct physical action, technical skill, and the navigation of often unpredictable human anatomy under pressure. The consequences of error can be immediate and irreversible. It is logical, therefore, that the surgical literature would be rich with discussions of pitfalls related to operative technique, instrumentation, anatomical variations, and the avoidance of iatrogenic injury.

In contrast, histopathology is a domain of interpretive and cognitive risk. The primary “procedure” is diagnostic interpretation. The pathologist confronts pitfalls related to visual perception, complex pattern recognition, and the cognitive biases that can lead to misinterpreting subtle morphological clues that differentiate benign from malignant conditions. The literature in this field is thus likely to focus on differential diagnoses, morphological mimics, and the cognitive traps that can lead to diagnostic error.

Finally, pediatrics represents a domain of developmental and systemic risk. The pediatric patient is a “moving target” physiologically, with constantly changing parameters for everything from drug metabolism to normal vital signs. Pitfalls in this field frequently relate to weight-based dosing calculations, recognizing diseases that present atypically in children compared to adults, and navigating the complex doctor–patient–parent communication triad. Therefore, the observed distribution of “pitfall” literature across specialties serves as a proxy map of the core epistemological and practical challenges that define the daily practice of these distinct medical disciplines.

The findings of this bibliometric analysis provide a quantitative foundation for understanding a significant, yet previously unmeasured, aspect of medical professional culture. The identification of over 37,000 publications dedicated to discussing “pitfalls” confirms that the proactive sharing of experiential knowledge about potential errors is a widespread and deeply embedded practice in modern medicine. This section will interpret the broader meaning of these results and discuss their implications for medical practice, education, and future research.

The sheer volume of literature identified in this study strongly supports the central thesis that these publications constitute a form of collective intelligence. The 37,295 articles represent a massive, peer-reviewed, and publicly accessible database of clinical wisdom. This is not a centrally planned or mandated system of reporting; it is a self-organizing phenomenon driven by what the data suggest is a perceived professional duty. The finding that physicians across all specialties are actively identifying, documenting, and publishing these error-prone situations demonstrates a powerful commitment to a shared goal: protecting colleagues from repeating mistakes and, by extension, protecting patients from harm.

This corpus functions as a vital supplement to traditional, evidence-based medicine. While randomized controlled trials and systematic reviews provide the “what” and “why” of clinical practice, the “pitfall” literature provides the crucial “how” and “what to watch out for.” It captures the tacit knowledge and hard-won experience that are difficult to quantify in a clinical trial but are essential for expert practice. It addresses the exceptions, the anomalies, and the contextual factors that formal guidelines often cannot. In this sense, the “pitfall” literature embodies the profession’s ongoing effort to make itself more resilient, adaptable, and self-correcting in the face of inherent uncertainty.

**Figure 1:**
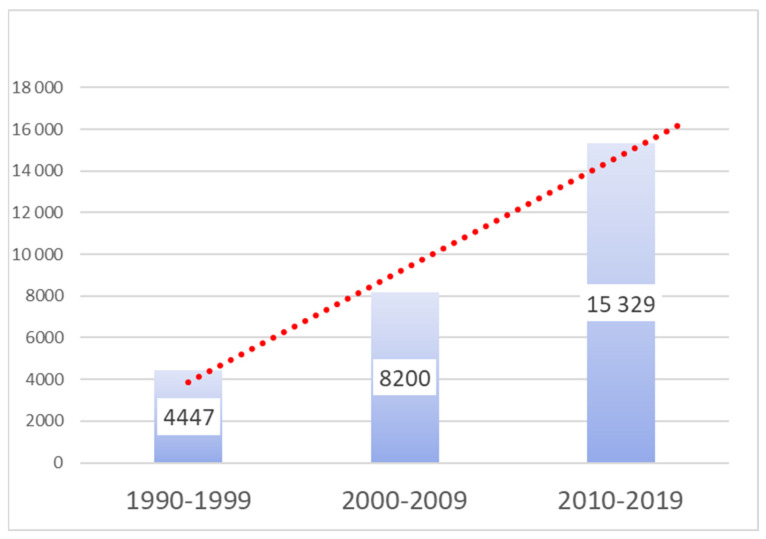
Trend of annual “pitfall” publications in PubMed (1990–2019). Source: Data derived from PubMed search. This figure illustrates the dynamic and expanding interest in clinical pitfalls, highlighting the growing relevance of this untapped knowledge base.

Perhaps more importantly, the trend of publishing on pitfalls is not static; it is accelerating. Figure 1 provides compelling visual evidence of a sustained and significant upward trend in the annual number of pitfall publications over the last three decades.

The data indicate a significant growth trend, with the number of publications on pitfalls roughly doubling each decade (e.g., from 4447 publications in 1990–1999 to 8/200 in 2000–2009 and 15,329 in 2010–2019). This demonstrates not only the sheer volume of this knowledge resource but also its increasing relevance and recognition within the medical community. Physicians are more actively identifying pitfalls and are increasingly aware of the need to report each discovered pitfall to their colleagues.

## 5. A Proposed Solution: The “Pitfall Bank” as a Proactive, Physician-Driven Prevention System

The existence of a vast and growing body of physician-reported pitfalls, combined with the failures of the top-down Safety-I paradigm, points to the need for a new prevention strategy. The primary barrier to leveraging this knowledge is not a lack of information, but a critical breakdown in knowledge translation. The data are abundant but inaccessible in the fast-paced clinical environment where they are needed most. We propose a solution to bridge this gap: the “Pitfall Bank”, a centralized, curated, online repository designed to function as a proactive, physician-driven error prevention system.

Conceptual synthesis was employed to develop the Pitfall Bank [18,19]. This research method primarily aims to integrate findings from multiple primary studies, as well as ideas, models, and theories drawn from diverse—often unrelated—fields, to produce a novel, cohesive conceptual framework or model. It constitutes an act of intellectual construction. The objective is not to provide an exhaustive survey of all existing literature within a given domain but rather to identify key ideas, models, and debates, and to critically examine their implications to address a specific problem or advance a new understanding.

The architecture of the Pitfall Bank is envisioned as a dynamic, user-friendly platform that consolidates all existing and newly emerging pitfall reports, systematizing them by specialty, condition, and error-prone situation. This system is designed to directly address the key challenges that currently prevent the widespread use of this experiential knowledge.

- Overcoming Inaccessibility and Time Constraints: Physicians at the point of care face immense time pressure. Studies indicate that while a comprehensive literature search can take nearly 30 min, most clinicians will abandon a search that lasts longer than two minutes [20]. The Pitfall Bank solves this “no time to search” problem by providing immediate, bedside access to relevant information. A physician facing a challenging diagnostic or therapeutic situation could simply enter the condition or scenario and receive a curated list of the most common pitfalls and the peer-vetted strategies to avoid them. This transforms the current state of being “unwarned and unarmed” against preventable errors into a state of being “forewarned and forearmed”.
- Fostering Collective Experience: Currently, physicians gain crucial experience in recognizing and avoiding pitfalls largely through individual trial and error—a long and often painful process that inevitably involves learning from mistakes that may harm patients. The Pitfall Bank fundamentally changes this dynamic by converting the scattered, individual experiences of thousands of physicians into a concentrated, collective intelligence. It allows a single physician to benefit from the hard-won wisdom of the entire medical community, compressing decades of individual learning curves into readily accessible insights. This accelerates the acquisition of expert-level situational awareness and directly reduces the patient harm that occurs during a clinician’s individual learning journey.
- Reducing Unwarranted Clinical Variation: The well-documented variability in diagnostic and treatment decisions among physicians for identical clinical situations is a significant quality and safety concern. This variation often indicates that, due to the limitations of individual experience, some clinicians may be struggling to identify the optimal path. The Pitfall Bank can serve as a platform for harmonizing best practices in high-risk scenarios. By providing access to a consensus view on how to navigate specific pitfalls, it can help reduce unwarranted variation and guide clinicians toward safer, more effective decisions.
- Bridging the Medical Education Gap: Modern medical education, both in universities and in Continuing Medical Education (CME) programs, largely focuses on teaching the standard methods for diagnosis and treatment. It often fails to systematically warn trainees about common errors and how to avoid them. The Pitfall Bank would serve as an invaluable educational resource, providing structured, practical, experiential knowledge of where things can go wrong. It could be integrated into training programs to better prepare the next generation of physicians for the real-world complexities of clinical practice.

## 6. The Pitfall Bank as a Translational Medicine Engine

The most novel and powerful aspect of the Pitfall Bank concept is its design not as a static repository, but as a dynamic, circular translational mechanism. The current stagnation in patient safety can be seen as a failure of translation—a breakdown in the connection between problems identified in clinical practice and the scientific development and implementation of solutions. Translational medicine provides a robust framework for repairing this connection, and the Pitfall Bank is designed to be the engine that drives this process for patient safety [21].

**Figure 2:**
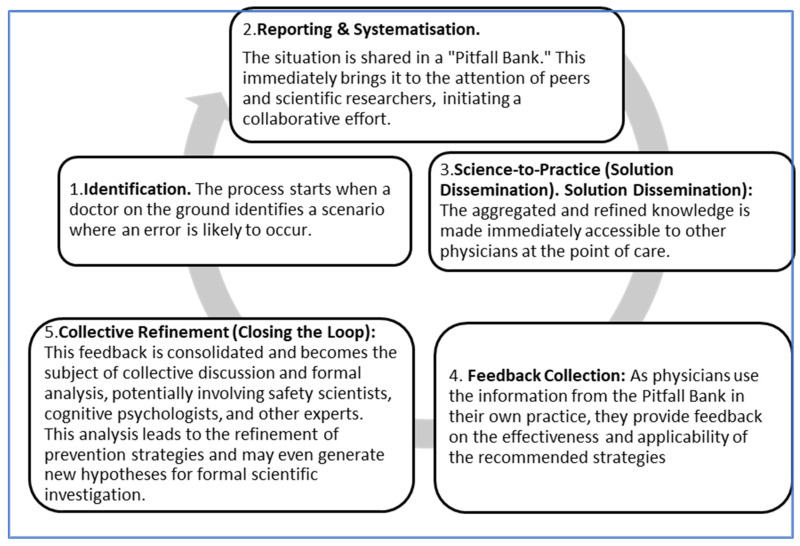
The “Pitfall Bank” translational feedback loop in medical error prevention. Source: Conceptual model developed by the authors. This figure illustrates the operating principle of the proposed “Pitfall Bank” as a dynamic, five-stage circular mechanism for the continuous improvement of medical error prevention, reflecting the principles of translational medicine.

Translational medicine is often conceptualized as a continuous cycle that moves observations from the “bedside” (clinical practice) to the “bench” (scientific research) and then translates the resulting scientific insights back into new interventions at the “bedside”. The Pitfall Bank operationalizes this cycle for the specific domain of experiential safety knowledge. The process is not merely an analogy; the system’s core components are explicitly designed to facilitate a continuous, five-stage feedback loop, as visualized in Figure 2.

The stages of this translational loop are as follows:

1. Identification of a risk-prone situation: The cycle begins with a practicing physician who identifies a high-risk situation or “pitfall” in their daily work. This frontline observation is the “bedside” data. The physician documents this pitfall and the context-specific strategies they used to avoid an error.
2. Reporting and Systematization: The physician’s report is submitted to the Pitfall Bank, where it is curated, peer-reviewed, and systematized alongside thousands of other reports. This step transforms isolated anecdotes into a structured, searchable knowledge base.
3. Science-to-Practice (Solution Dissemination): The aggregated and refined knowledge is made immediately accessible to other physicians at the point of care. This is the “bench-to-bedside” transfer, where the collective wisdom of the community is delivered as a practical tool to prevent harm.
4. Feedback Collection: As physicians use the information from the Pitfall Bank in their own practice, they provide feedback on the effectiveness and applicability of the recommended strategies. This creates a new layer of real-world data on the intervention’s performance.
5. Collective Refinement (Closing the Loop): This feedback is consolidated and becomes the subject of collective discussion and formal analysis, potentially involving safety scientists, cognitive psychologists, and other experts. This analysis leads to the refinement of prevention strategies and may even generate new hypotheses for formal scientific investigation. This closes the translational loop, initiating a new cycle of continuous improvement.

By creating this engine, the Pitfall Bank moves beyond being a simple information resource. It becomes a living system for collaborative learning and improvement, integrating practical experience with scientific rigor to solve the most challenging problems in patient safety.

## 7. Discussion

The proposal for a physician-driven Pitfall Bank represents a significant departure from prevailing patient safety paradigms. This section synthesizes the principal findings, evaluates the novelty of the proposed model relative to existing systems, explores its broader implications for policy and practice, and acknowledges the limitations and future research directions of this conceptual work.

### 7.1. Principal Findings and Contributions

The central argument of this paper is that a quarter-century of efforts to reduce medical errors has reached an impasse due to an overreliance on the reactive, top-down Safety-I paradigm. This approach is ill-suited to the complexity of modern medicine, hampered by flawed data, and neglectful of clinician adaptation as a source of resilience.

Through a quantitative bibliometric analysis, this review identified a vast, growing, and systematically underutilized resource: more than 43,000 physician-authored publications on “clinical pitfalls.” The principal contribution of this work is the conceptualization of the Pitfall Bank—a novel system designed to harness this untapped knowledge. Its strength lies in grounding itself in Safety-II principles, which position clinicians as a resource for resilience, and in its architecture as a circular translational mechanism. This design directly addresses the core failures of Safety-I by creating a proactive, bottom-up, continuously learning system that bridges the gap between frontline clinical experience and evidence-based practice.

### 7.2. Comparison with Existing Systems and Novelty of the Proposed Model

Although the sharing of safety information is not new, the Pitfall Bank differs from existing systems in several fundamental ways:

- Reactive vs. Proactive Focus: Existing official databases, such as the FDA’s MedWatch and the Manufacturer and User Facility Device Experience (MAUDE) database, are inherently reactive. They catalogue adverse events and device failures after they occur [22,23]. By contrast, the Pitfall Bank is proactive, focusing on identifying and disseminating knowledge about high-risk situations before they result in harm, shifting emphasis from incident analysis to risk anticipation.
- Top-Down vs. Bottom-Up Knowledge Generation: Many safety initiatives, including some physician-led efforts, operate top-down—relying on expert panels to develop guidelines or protocols for specific error types, such as medication administration or surgical procedures [24,25]. While valuable, these approaches fail to capture the full spectrum of frontline experiential knowledge. The Pitfall Bank is inherently bottom-up, driven by the collective, real-world experiences of practicing physicians.
- Static vs. Dynamic Systems: Traditional clinical research databases and case repositories are typically static collections used for retrospective analysis or teaching [26,27]. Similarly, online physician forums, while fostering discussion, are unstructured and lack mechanisms for systematic curation and refinement. The Pitfall Bank is dynamic, functioning as a living system with a continuous translational feedback loop (Figure 2) in which knowledge is submitted, refined through peer feedback, and reintegrated into practice. This circular, self-improving process constitutes its most significant innovation.

The synthesis of the main advantages of the Pitfall Bank is presented in Table 4.

### 7.3. Implications for Policy, Practice, and Economic Viability

Implementation of a Pitfall Bank would have far-reaching implications that extend beyond clinical practice to encompass cultural change, professional development, and profound economic benefits for the healthcare system.

#### 7.3.1. Fostering a Just Culture and Enhancing Professional Development

The primary value of the Pitfall Bank is not merely informational but cultural. It serves as a powerful mechanism for operationalizing a “just culture”, a concept that is foundational to meaningful progress in patient safety [28]. The dominant Safety-I paradigm, with its focus on identifying and eliminating failures after they occur, has inadvertently fostered a “culture of blame” that discourages open communication about risk. Clinicians, fearing reputational harm or litigation, are often reluctant to report near-misses or discuss error-prone situations, leading to chronic underreporting and a distorted view of systemic vulnerabilities. The Pitfall Bank directly subverts this dynamic. By shifting the focus from individual culpability for past failures to collective, proactive learning from high-risk scenarios, it creates a psychologically safe environment for clinicians to share vital experiential knowledge without fear of reprisal.

This practitioner-driven approach also offers a potent solution to the persistent problem of unwarranted clinical variation. Such variation often signals that clinicians, relying on disparate levels of individual experience, are struggling to identify the optimal path in complex situations. The Pitfall Bank facilitates the emergence of a professionally led consensus on best practices for navigating specific, high-risk scenarios. This bottom-up method of harmonizing practice is far more likely to gain traction and be adopted by clinicians than administratively imposed, top-down mandates.

Finally, the platform functions as an invaluable, on-demand resource for continuous professional development and lifelong learning. Modern medical education often excels at teaching standard procedures but frequently fails to systematically warn trainees about common pitfalls and how to avoid them. The Pitfall Bank bridges this critical gap between “work-as-imagined” in textbooks and the complex, adaptive realities of “work-as-done” at the bedside, accelerating the acquisition of expert-level situational awareness.

#### 7.3.2. The Economic Imperative: A Return-on-Investment Case for the Pitfall Bank

While the conceptual proposal for the Pitfall Bank faces practical challenges, particularly that of securing sustainable funding, this concern can be decisively addressed by reframing the financial calculus. Viewing the Pitfall Bank as a mere operational cost is a fundamental mischaracterization. A more accurate and strategic evaluation frames it as a high-yield investment in system resilience, safety, and financial stability. The most appropriate analytical lens for this evaluation is the Return on Investment (ROI) model, a framework well understood by healthcare administrators, policymakers, and institutional leadership [29]. An ROI analysis provides a formal methodology to calculate the net financial gains of an intervention by comparing the resources invested against the returns generated through cost-avoidance [30,31]. When applied to the Pitfall Bank, this analysis reveals that the economic argument for its implementation is not just viable but overwhelming.

To fully appreciate the potential return, one must first comprehend the staggering cost of inaction—the price the healthcare system currently pays for its systemic vulnerabilities. This price is not a single figure but a multi-layered cascade of direct, liability-related, and systemic costs.

First, the direct medical costs associated with managing the consequences of preventable adverse events are immense. Conservative estimates place the annual cost of measurable medical errors in the United States between USD 17.1 billion and USD 20 billion [32,33]. These figures account for the additional resources consumed by extended hospitalizations, repeat or remedial procedures, and the complex management of complications such as hospital-acquired infections, which alone are estimated to add between $35.7 billion and $45 billion to annual healthcare spending. These are direct, tangible losses that represent a profound misallocation of healthcare resources.

Second, the financial burden of medical liability and litigation is substantial. The average indemnity payment for a medical malpractice claim that results in a payout now exceeds USD 350,000, with the average jury verdict for plaintiffs surpassing USD 1 million [34]. Critically, these figures do not capture the full scope of litigation-related expenses. Significant costs are incurred even when a claim is ultimately determined to be without merit; the average expense to defend a claim that is dropped, dismissed, or withdrawn is over USD 30,000. These expenditures represent a direct and quantifiable drain on healthcare resources stemming from the very types of preventable errors and high-risk situations the Pitfall Bank is designed to mitigate.

Third, and perhaps most insidiously, are the systemic costs rooted in the prevailing Safety-I “culture of blame.” This punitive environment is a well-documented driver of physician burnout, a crisis with devastating economic consequences. Physician burnout is not merely a matter of professional dissatisfaction; it is estimated to cost the U.S. healthcare system USD 4.6 billion annually, which translates to approximately USD 7600 per physician per year, primarily through physician turnover and reduced clinical work hours [35,36]. The cost to an individual healthcare organization to replace a single physician is staggering, ranging from USD 500,000 to over USD 1 million when accounting for recruitment, signing bonuses, onboarding, and lost billings during the transition period. Furthermore, this turnover has downstream effects on the entire system, adding nearly USD 1 billion in excess health care expenditures for patients annually due to disruptions in care continuity, which lead to increased use of more expensive specialty and emergency services.

These three domains of cost are not independent variables; they are locked in a pernicious and self-perpetuating economic feedback loop. The Safety-I paradigm’s focus on blame and punishment directly contributes to the high rates of physician burnout. Burnout, in turn, is strongly associated with an increased likelihood of making medical errors and a decrease in patient satisfaction. This rise in errors leads directly to more adverse events, inflating the direct medical costs, and simultaneously increases the risk of malpractice litigation. The heightened fear of litigation then reinforces the very “culture of blame” that initiated the cycle, creating a vicious loop that continuously amplifies systemic costs. An intervention that can break a single link in this chain—for instance, by supplanting the culture of blame with a psychologically safe, non-punitive learning environment—will have cascading positive financial effects across all other domains. The Pitfall Bank, by its very design, is precisely such an intervention.

A final, crucial economic consideration is the distribution of these costs. A significant barrier to institutional investment in safety has been the fact that hospitals do not bear the full financial consequences of their errors. Studies have shown that a large proportion of the costs of adverse events—up to 78%—are externalized to other parties, including public and private payers, and patients themselves. This reality can weaken the internal “business case” for safety investments if the argument is based solely on avoiding direct medical costs. However, the economic case for the Pitfall Bank powerfully overcomes this disincentive. While direct error costs may be partially externalized, the substantial costs associated with physician burnout and turnover are borne almost entirely by the healthcare organization itself. The expenses of recruitment, training, and lost productivity are direct, non-externalizable hits to the institution’s bottom line. Because the Pitfall Bank, by fostering a just culture and reducing the psychological strain of practice, is a direct intervention against the primary drivers of burnout, its most compelling ROI is arguably in human capital preservation. From a CFO’s perspective, an investment in a Pitfall Bank program that prevents the turnover of even a single physician can be cost-neutral or even highly profitable, entirely independent of its effects on adverse events or payer reimbursement models.

Therefore, the Pitfall Bank is not an expense to be minimized but a high-leverage investment to be prioritized. Its ROI is generated through significant cost-avoidance across multiple, interconnected domains. Preventing even one high-cost adverse event or retaining one physician who might otherwise have left due to burnout can offset the program’s operational costs for years. The following Table 5 provides a consolidated view of this powerful economic case, juxtaposing the price of inaction with the projected returns from a strategic investment in this physician-driven, proactive safety system.

### 7.4. Limitations and Future Research

As a conceptual proposal, this work faces several limitations. Chief among them are the practical challenges of implementation, including securing sustainable funding, establishing robust governance and moderation, developing user-friendly technological infrastructure, and—most critically—motivating sustained engagement from busy clinicians. These challenges point to several priorities for future research. The immediate next step should be to design and evaluate a pilot Pitfall Bank within a defined specialty or healthcare system, assessing feasibility, clinician engagement, and preliminary impact. Additional studies should investigate the cognitive mechanisms through which pitfall warnings influence decision-making and reduce error rates. Future research could also explore expanding the concept to include other healthcare professions, such as nursing and pharmacy, and extending its scope to address pitfalls in interprofessional communication and organizational processes—both major contributors to medical error.

## 8. Conclusions

The rapid advancement and increasing complexity of modern medicine are accompanied by a corresponding rise in the number and diversity of error-prone clinical situations. The prevailing error prevention paradigm—rooted in Safety-I principles and dependent on standardized, centrally mandated measures—has proven insufficient to meet this challenge and has failed to meaningfully reduce the incidence of medical errors. Persisting with this approach, while the complexity of care continues to accelerate, risks a sharp escalation in patient harm.

A fundamental paradigm shift is required. A physician-driven pitfall-reporting system, grounded in the proactive and resilience-oriented principles of Safety-II, directly addresses the limitations of current prevention methods. By harnessing the vast body of collective experiential knowledge from frontline clinicians, such a system facilitates rapid, context-specific interventions and cultivates a dynamic, shared intelligence for error prevention. When designed as a circular translational mechanism, the proposed Pitfall Bank becomes more than a database: it functions as both a sensitive surveillance tool and a continuous improvement engine. It enables an iterative cycle of identifying emerging error-prone situations, alerting physicians to them, developing and disseminating prevention strategies, synthesizing the results of their application, and refining these measures for reintegration into practice.

In light of the overwhelming evidence presented, the implementation of such a system is not only an ethical necessity for patient safety but also a fiscal imperative for the long-term sustainability of the healthcare system. The multi-billion-dollar annual costs of preventable errors, litigation, and physician burnout demonstrate that the failure to invest in proactive, clinician-empowering systems like the Pitfall Bank represents a far greater financial risk than the cost of their implementation. Given the dynamic nature of contemporary medical practice, this physician-driven, translational approach offers the most promising strategy for sustaining medical progress while effectively mitigating associated risks—ultimately fostering a safer, more resilient, and economically sound healthcare system.

## Figures and Tables

**Table 1:** Foundational paradigms in patient safety: Safety-I vs. Safety-II.

Dimension	Safety-I	Safety-II
Definition of Safety	A state where the number of adverse outcomes is as low as possible (freedom from unacceptable harm).	A state where the number of successful outcomes is as high as possible (the ability to succeed under varying conditions).
View of Error	Errors are caused by failures of components (technical or human) and represent a deviation from required performance.	Errors are the inevitable downside of normal performance variability; the same adaptations that usually create success can, in certain conditions, lead to failure.
Prevention Strategy	Reactive: Find and fix the causes of adverse events. Eliminate or constrain human variability.	Proactive: Understand how everyday work succeeds. Enhance the system’s ability to adapt and be resilient.
Role of Clinician	A potential source of error and liability whose performance must be controlled and monitored through rigid protocols.	A critical resource for resilience and safety whose adaptive capacity is necessary for the system to cope with complexity.
Application in Healthcare	Top-down administrative mandates (e.g., surgical checklists, CPOE) effective for simple, linear tasks.	Bottom-up, clinician-driven initiatives (e.g., the proposed “Pitfall Bank”) that leverage experiential knowledge for complex, dynamic tasks.

Source: Synthesized from Hollnagel et al. and other safety science literature [8,9].

**Table 2:** PubMed advanced search query construction.

Query Component	Search String Example	Field	Rationale/Purpose
Core Term Search (General)	“pitfall” [All Fields] OR “pitfalls” [All Fields]	[All Fields]	To capture any mention of the singular or plural form of the term anywhere in the publication record (including full text where available).
Core Term Search (Title)	“pitfall” OR “pitfalls” AND [Title]	[Title]	To identify publications where the discussion of pitfalls is the primary topic, as indicated by its inclusion in the article title.
Core Term Search (Title/Abstract)	“pitfall” OR “pitfalls” AND [Title/Abstract])	[Title/ Abstract])	To identify publications where pitfalls are a key theme, important enough to be mentioned in the title or the author-supplied summary.
Specialty Modifier	“Surgery” [All Fields]	[Specialty]	To filter the specialty.
Date Filter	1 January 1990:28 February 2024	[pdat]	To limit the search to the defined period of modern medicine, from 1 January 1990 to the end of the last full month before the search date.

**Table 3:** Number of publications referencing “pitfalls” by select medical specialties (1990–2024).

Medical Specialty	Total Publications with “Pitfall”	Publications with “Pitfall” in Title	Publications with “Pitfall” in Abstract	% of Total with “Pitfall” in Title or Abstract
Surgery	9960	1152	1971	19.8%
Histopathology/Pathology	8940	1661	2772	31.0%
Radiology	5587	694	1129	20.2%
Pediatrics	2064	139	249	12.1%
Internal Medicine	1339	119	228	17.0%
Laboratory Medicine	1765	219	389	22.0%
Anesthesiology	360	15	27	7.5%
Endocrinology	543	47	75	13.8%
Ophthalmology	277	1	1	0.4%
All Specialties	37,295	3044	6249	16.8%

**Table 4:** Synthesis of the main advantages of the Pitfall Bank.

Identified Failure in Current System (Safety-I)	Proposed Solution via “Pitfall Bank” (Safety-II)
Lack of Hazard Recognition: Doctors may not recognize that a situation is high-risk, so they fail to take preventive measures.	Targeted Alerts: Physician-driven, context-specific warnings that address knowledge gaps and trigger analytical (System 2) thinking.
Siloed Experience: Each physician individually develops their experience in identifying and preventing high-risk situations. This experience is therefore subjective and limited to their personal practice.	Collective Intelligence: Individual experiences are integrated into a collective knowledge base, made easily accessible through the Pitfall Bank.
Slow Learning Curve: Acquiring the experience to identify and prevent high-risk situations is a slow process for individual doctors, often requiring years of practice and learning from personal mistakes.	Accelerated Expertise: The collective experience curated by the Pitfall Bank is available to physicians from the beginning of their careers, helping to prevent errors.
Unreliable Error Data: A “culture of blame” and fear of litigation lead to the chronic underreporting of adverse events.	Psychological Safety: A non-punitive, educational platform focused on proactive learning from high-risk situations, not reactive reporting of failures.
Gap Between Research and Practice: Lack of a functional mechanism to connect frontline problems with scientific solutions.	Translational Feedback Loop: A circular feedback system that systematically bridges the gap between clinical practice and scientific research.
Inaccessibility of Knowledge: Experiential knowledge is scattered across thousands of publications, making it unusable at the point of care due to time constraints.	Knowledge at Your Fingertips: A centralized, curated, and instantly searchable repository that delivers relevant, actionable knowledge to the bedside in seconds.

**Table 5:** Cost-effectiveness of the Pitfall Bank: a return-on-investment analysis.

Domain of Economic Impact	Current Annual Costs (The Price of Inaction)	Projected Impact of the Pitfall Bank (Return on Investment)	Supporting Evidence and Data
1. Direct Medical Costs of Preventable Adverse Events	USD 17.1–USD 20 billion annually in the U.S. from measurable medical errors. These costs arise from additional care, extended hospitalizations, and managing complications.	Cost-Avoidance: By proactively warning clinicians of high-risk scenarios, the Pitfall Bank reduces the incidence of preventable errors, leading to direct savings from fewer complications, shorter lengths of stay, and reduced need for remedial procedures.	[32,33]
2. Medical Liability and Litigation	Average malpractice indemnity payment exceeds USD 350,000. The average cost to defend a claim, even if dismissed, is >USD 30,000.	Risk Mitigation: The platform serves as evidence of a proactive institutional commitment to safety. By arming physicians with peer-vetted knowledge to avoid common pitfalls, it directly reduces the frequency of negligent errors, thereby lowering the incidence of costly claims and substantial defense expenditures.	[34]
3. Systemic Costs of Physician Burnout and Turnover	USD 4.6 billion annually in national costs attributable to physician burnout. The direct cost to an organization to replace a single physician is USD 500,000–USD 1,000,000+.	Human Capital Preservation: By fostering a non-punitive “just culture” (a Safety-II principle), the Pitfall Bank directly addresses a primary driver of burnout. This improves physician retention, avoiding massive, non-externalizable turnover costs and preserving productivity and institutional knowledge.	[35,36]

## Data Availability

The publications used for this bibliometric study were retrieved from PubMed. The research methodology is presented in Section 4 of this article. Table 2 outlines the components of the search formulas, and the results are summarized in Table.
